# Functional Neuronal Differentiation of Injury-Induced Muscle-Derived Stem Cell-Like Cells with Therapeutic Implications

**DOI:** 10.1038/s41598-017-01311-4

**Published:** 2017-04-26

**Authors:** Kinga Vojnits, Haiying Pan, Xiaojing Dai, Hao Sun, Qingchun Tong, Radbod Darabi, Johnny Huard, Yong Li

**Affiliations:** 1 Center for Stem Cell and Regenerative Medicine, The Brown Foundation Institute of Molecular Medicine for the Prevention of Human Diseases (IMM) at the University of Texas Health Science Center at Houston, TX Houston, 77030 USA; 2 Department of Pediatric Surgery, University of Texas McGovern Medical School at Houston, Houston, 77030 TX USA; 3 Department of Orthopeadic, University of Texas McGovern Medical School at Houston, Houston, 77030 TX USA; 4 Center for Metabolic and Degenerative Disease, The IMM at the University of Texas Health Science Center at Houston, TX Houston, 77030 USA; 5 Center for Sports Regenerative Medicine, Steadman Philippon Research Institute, Vail, CO USA; 6 Center for Tissue Engineering and Aging Research, The IMM at the University of Texas Health Science Center at Houston, TX Houston, 77030 USA

## Abstract

Mammalian skeletal muscles contain a number of heterogeneous cell populations. Our previous study characterized a unique population of myogenic lineage stem cells that can be isolated from adult mammalian skeletal muscles upon injury. These injury-induced muscle-derived stem cell-like cells (iMuSCs) displayed a multipotent state with sensitiveness and strong migration abilities. Here, we report that these iMuSCs have the capability to form neurospheres that represent multiple neural phenotypes. The induced neuronal cells expressed various neuron-specific proteins, their mRNA expression during neuronal differentiation recapitulated embryonic neurogenesis, they generated action potentials, and they formed functional synapses *in vitro*. Furthermore, the transplantation of iMuSCs or their cell extracts into the muscles of *mdx* mice (i.e., a mouse model of Duchenne Muscular Dystrophy [DMD]) could restore the morphology of their previously damaged neuromuscular junctions (NMJs), suggesting that the beneficial effects of iMuSCs may not be restricted to cell restoration alone, but also due to their transient paracrine actions. The current study reveals the essential role of iMuSCs in the restoration of NMJs related to injuries and diseases.

## Introduction

Under normal conditions, skeletal muscle can repair itself by removing damaged myofibers and synthesizing new muscle fibers to restore functional contractile properties^1^. In line with its regenerative property, skeletal muscle is enriched with stem cells^2^. The resident satellite cells and muscle stem cells (MuSCs), which are populations of mononucleated cells located between the basal lamina and sarcolemma of muscle fibers, are responsible for the postnatal growth, repair, and maintenance of skeletal muscle^3^. After necrosis of damaged muscle fibers, an inflammatory response is initiated which leads to the phagocytosis of injured myofibers and the activation of normally quiescent MuSCs^4–6^. The activated MuSCs proliferate, migrate to the site of injury, fuse, and differentiate to form new myofibers^7^. In the last few years, researchers have shown that MuSC transplantation is a promising tool for both the repair and regeneration of skeletal muscle tissues. However, their loss of ‘stemness’ during culture, their inability to cross the vessel wall for systemic delivery, and their poor survival after implantation greatly compromise their therapeutic efficacy^8, 9^.

Recent studies have discovered that skeletal muscles contain a number of heterogeneous cell populations^10, 11^. Several stem cell-like cells (including MuSCs), various side populations^12^, muscle progenitor cells^13^, and putative myoendothelial precursors^14^ have been identified in skeletal muscle tissues based on their expression of surface markers. These cells displayed multipotency and can differentiate into other lineages, such as ectodermal neuronal cells^15^. Previous studies have been limited to MuSCs derived from healthy, uninjured muscles. In fact, following muscle injury, the local microenvironment of resident precursor cells become altered^16, 17^ which can lead to changes in their phenotype and biomolecular characteristics. Our recent studies^18^ have shown that a unique population of MuSCs, named iMuSCs exist in injured murine skeletal muscle, and can be isolated by using a modified preplate technique^19–21^ and a Cre-LoxP system that established in our laboratory^22^. This unique population of iMuSCs expressed several pluripotent and myogenic stem cell markers, such as Oct4 (also called as Pou5fl), Sox2 (SRY-box 2), Nanog, Msx1 (Msh homeobox 1), Sca1 (Stem cell antigen-1), Pax7 (Paired box protein 7), and CD34^18^. When compared to MuSCs isolated from uninjured muscles, iMuSCs were extremely sensitive to transient microenvironmental changes, had elevated migratory capacity, and had strong myogenic properties both *in vitro* and *in vivo* ^20^. We also found that these iMuSCs were capable of differentiating into non-myogenic related lineages (such as CD31^+^ endothelial-like cells^20^) and fulfilled several *in vitro* and *in vivo* criteria for multipotency^18^. These results strongly suggest that the stimulation of injuries can reprogram iMuSCs to a more multipotent state while maintaining their myogenic origin.

Of particular interest is the reported ability of iMuSCs to differentiate into neural lineages *in vitro* ^18^. In the current study, we investigated the regenerative potential of iMuSCs with specific focus on neurogenesis*. In vitro*, we have studied neuronal-like cells developed from iMuSCs, and *in vivo*, we evaluated the efficiency of iMuSCs in repairing neuromuscular junctions (NMJs) following intramuscular transplantation into *mdx* mice, a murine model that represents Duchenne Muscular Dystrophy (DMD).

## Materials and Methods

### Animal studies

All animal experiments and related experimental protocols were approved by the Center for Laboratory Animal Medicine and Care at The University of Texas Health Science Center at Houston. The methods were carried out in accordance with the approved guidelines. Female *C57BL/6J* mice and male *mdx* mice were used in this study (Jackson Lab; Bar Harbor, ME, USA). Muscle injuries were created following previously published protocols^20^. Briefly, the tibialis anterior (TA) muscle in one leg of each mouse (female, 4–8 weeks-old, *C57BL/6J*) was lacerated^23^, and the TA muscle in the other leg of the same donor mouse served as the control uninjured muscle.

### Cell isolation and culture

Four days after laceration injury, the modified preplate technique was used to isolate MuSCs from the uninjured TA muscles and iMuSCs from the injured TA muscles of *C57BL/6J* mice^18, 19, 21^. By utilizing this technique, different cell populations could be obtained based on their cell adhesion characteristics: fast adhering fibroblast-like cells, myoblasts, and slow adhering MuSCs. iMuSCs were separately cultured in ESGRO Complete PLUS Clonal Grade Medium (Millipore, USA) in 12-well tissue culture plates (Corning, USA) for 3 weeks^18^. The medium was then replaced with normal muscle growth medium^21^ and iMuSCs were further cultured and expanded on collagen type IV-coated flasks in 5% CO_2_ at 37 °C. The control MuSCs were cultured at the same time on collagen type IV-coated flasks in normal muscle growth medium in 5% CO_2_ at 37 °C.

### *In vitro* differentiation via neurosphere formation

Undifferentiated iMuSCs were cultured in suspension in Neural Stem Cell (NSC) medium [Neurobasal medium (Invitrogen, USA) supplemented with 1% Glutamax, 1% B-27 Supplement (Invitrogen, USA), 20 ng/ml basic fibroblast growth factor, and 0.5% Penicillin/Streptomycin antibiotics; unless otherwise mentioned, all from Gibco (USA)] for 1 week. To induce neurogenic differentiation, neurospheres were plated on collagen type IV/Poly-L-ornithine/Laminin (USA) coated 24-wells plates or on Poly-L-ornithine/Laminin coated glass coverslips and cultured for 21 days in Neural Differentiation (ND3) medium [Neurobasal medium (Invitrogen, USA) supplemented with 1% Glutamax (Gibco, USA), 1% B-27 Supplement (Invitrogen, USA), 20 ng/ml brain-derived neurotrophic factor (Sigma, USA), and 0.5% Penicillin/Streptomycin (Gibco, USA).

**Figure 1:**
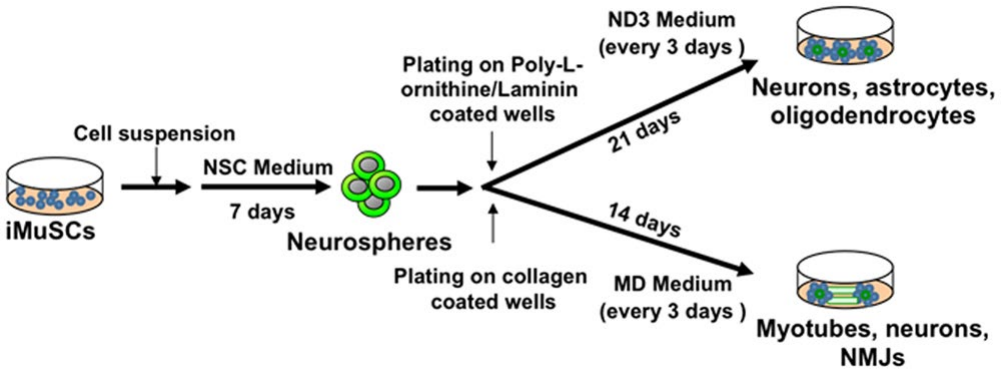
*In vitro* differentiation protocol. Undifferentiated iMuSCs were cultured in Neural Stem Cell (NSC) Medium in suspension for 7 days to induce neurosphere formation. For myotube differentiation, neurospheres were plated on collagen-coated wells in Muscle Differentiation (MD) Medium. For neural differentiation, neurospheres were plated on laminin/poly-L-ornithine coated wells and cultured for 21 days in Neural Differentiation (ND3) Medium.

Additionally, for myotube differentiation, neurospheres were plated on collagen type IV coated wells in Muscle Differentiation (MD) medium [DMEM supplemented with 2% horse serum (HS) and 1% Penicillin/Streptomycin antibiotics (Gibco, USA) and cultured for 14 days (Fig. 1).

### Immunocytochemistry

Samples were fixed with 4% paraformaldehyde (Sigma, USA) and were then permeabilized for 30 minutes with 0.2% Triton X-100 (Sigma, USA). Nonspecific binding of antibodies was blocked with 10% bovine serum albumin (BSA) and 5% HS (Sigma, USA) for 1 hour. The primary antibodies were applied overnight at 4 °C then incubated for 1 hour at room temperature with the appropriate fluorescent-conjugated secondary antibodies (Suppl. Table 1). The nuclei were visualized using 4′,6′-diamidino-2-phenylindole (DAPI) staining (Sigma, USA) and fluorescent microscopy (Nicon). The resulting images were quantitatively analyzed using ImageJ Software (National Institutes of Health, USA).

### Quantitative real-time PCR

Total RNA from the MuSCs, iMuSCs, and the neuronal differentiating iMuSCs were isolated using the RNeasy Plus Mini Kit (Qiagen, USA). Total RNA from the biopsied TA tissues was isolated using the RNeasy Microarray Tissue Mini Kit (Qiagen, USA). cDNA was synthesized from 1 µg of RNA via iScriptTM cDNA Synthesis Kit (Bio-Rad, USA) following manufacturer’s instructions. Gene expression was analyzed by quantitative real-time PCR (qPCR) using MyiQ real-time PCR (Bio-Rad, USA). The applied primers were designed by Oligo software (Suppl. Table 2) (Oligo Perfect Designer, Invitrogen, USA). Reactions were measured in duplicate using custom 2x Syber Green Master Mix based on the hot-start Jumpstart Taq DNA Polymerase enzyme (Sigma). The amplification was done for 40 cycles (95 °C 20 sec, 60 °C 20 sec, 72 °C 40 sec). To verify the PCR product, melting curves were carried out in each reaction. Relative quantification of mRNA was determined by the ΔΔCt method (2^−ΔΔCt^ formula)^24^ using the expression profile of the corresponding control samples as reference.

### Whole-cell patch-clamp recording

Electrophysiological recordings were performed in whole-cell recording configuration under voltage and current-clamp mode. Patch pipettes were pulled from glass capillaries with an outer diameter of 1.5 mm on a Micropipette Puller (P-97, Sutter Instrument, Novato, CA, USA). The resistance between the recording electrode filled with pipette solution and the reference electrode was 4–6 MΩ. Membrane current and voltage signals were measured using a patch-clamp amplifier (Axon 700B, Axon Instruments, Sunnyvale, CA, USA), were sampled using a Digidata 1440 interface connected to a personal computer, and were analyzed with Clampex and Clampfit software (Version 10.3, Axon Instruments). In most experiments, 70% series resistance was compensated. The membrane potential was held at −70 mV during voltage-clamp mode. All experiments were carried out at room temperature (22–25 °C).

The standard external solution contained (in mM) 150 NaCl, 5KCl, 1 MgCl_2_, 2 CaCl_2_, 10 glucose, and 10 HEPES (4-(2-hydroxyethyl)-1-piperazineethanesulfonic acid). The pH was adjusted to 7.4 with Tris base. The osmolality of the solutions was adjusted to 310–320 mOsm/L with sucrose and a microosmometer (Advanced Instruments, Inc., Norwood, MA, USA; Model 3300). The pipette solution for whole-cell patch-clamp recording contained (in mM) 90K^+^-gluconate, 40 KCl, 1 MgCl_2_, 10 NaCl, 10 EGTA (ethylene glycol tetraacetic acid), 4 Mg-ATP, and 10 HEPES/KOH (pH 7.4). Whole-cell recordings were obtained from neuronal differentiated iMuSCs at day 21. The protocol for voltage clamp recording of Na^+^ channels was as follows: cells were hyperpolarized to −90 mV, stepped to a defined voltage as indicated, and returned to −70 mV before the next cycle was run (with a different voltage step). Each cycle took 120 ms. For K^+^ current recording, current-voltage (I-V) relationships of membrane currents were recorded using a ramp protocol (−100 to +50 mV). Na^+^ and K^+^ currents were blocked by tetrodotoxin (1 µM) and tetraethylammonium (5 mM) separately. In the current clamp, action potentials were generated by current injections of increasing magnitude with a duration of 600 ms (10 pA steps, from −20 pA to +90 pA). Spontaneous action potentials were also recorded in current clamp mode (0 pA).

### Transplantation of iMuSCs into mdx mice

Both control MuSCs and iMuSCs were pre-labeled with GFP (1 × 10^6^ cells diluted in 10 μL PBS) and were separately injected into the left and right gastrocnemius (GM) muscles of five 6- to 8-week-old male *mdx* mice. Additionally, in other experiments, cell extracts prepared from iMuSCs and MuSCs were separately injected into the left and right GM muscles of five 6- to 8-week-old *mdx* mice. iMuSCs, MuSCs, and PBS-treated GM tissues were harvested from each mouse for histological and qPCR analysis both 1 and 3 weeks after transplantation.

### Assessment of NMJ morphology

Harvested muscle tissues were mounted and frozen in cooled 2-methylbutane immersed in liquid nitrogen. Each muscle sample was cryosectioned to a thickness of 30 μm, fixed with 4% paraformaldehyde, and permeabilized. Nonspecific binding of antibodies was blocked with 10% HS, and Alexa-594 conjugated α bungarotoxin (Btx) (diluted 1:1000 in 10% HS/PBS) was applied for 1 hour at room temperature. Results were visualized by Leica TCS SP5 confocal microscopy.

### Data processing and statistical analyses

A minimum of five biological replicates was established for each of the described experiments. qPCR expression data was analyzed by MyiQ qPCR software (Bio-Rad, USA). The Ct values were exported and used as raw data for analysis of qPCR data. Data normalization was calculated using endogenous control β-actin and GAPDH, and fold changes were determined by the ΔΔCt method (2^−ΔΔCt^)^24^ by using the expression profile of the corresponding control as reference. Statistical analyses were carried out using GraphPad Prism version 6.0 (Graph Pad Software, Inc, USA). All numerical data were expressed as mean values ± SD. Comparisons between two groups were performed by using Student’s t-test assuming two-tailed distribution, and unequal variances. For multiple comparisons, ANOVA or Kruskal-Wallis test was applied. Statistical significance was considered at p < 0.05.

In order to account for the depth of NMJs during the assessment of NMJ morphology, a maximum intensity flat plane projection was made from Z-stacked images using the ImageJ software. Only NMJs in a complete “*en face* view” was selected for further analysis. After corrections were made to account for background noise, binary images were created, skeletonized, and histograms describing the connectivity for each pixel were generated. The number of segments described the density of NMJs and the means of terminal and branching nodes were also calculated by the Amira software (Fei, USA). At least 15 images were blindly selected and analyzed and five biological replicates were used for the study. The number of clusters within individual NMJs was counted using unprocessed images.

## Results

### Neurosphere, myotube, and NMJ formation of iMuSCs

With our successful cell isolation method^15, 20, 22^, iMuSCs were isolated from the slow adhering CD34^+^/Sca1^+^ fraction from the injured murine TA muscles and expanded in culture in muscle growth medium. As we have previously reported^18^, two weeks after cell isolation, proliferating iMuSCs (~0.1% of the entire muscle cell population) appeared in the culture dishes in the ESGRO Complete PLUS Clonal Grade Medium; however, no such cells were detected in the cultures established from control, uninjured muscles^18^.

**Figure 2:**
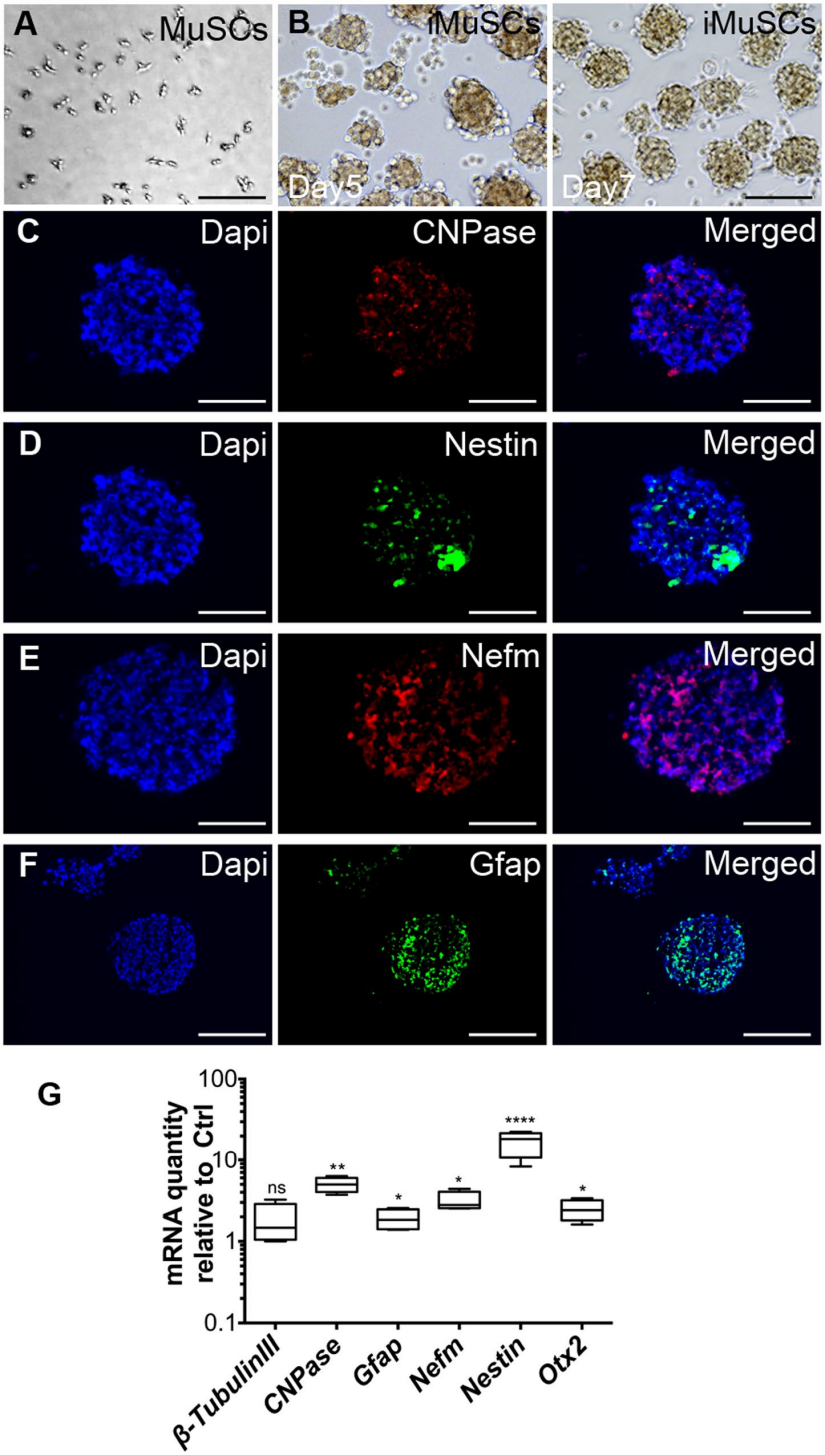
Neurosphere formation of iMuSCs. (**A**) BF image of the control MuSCs that could not form neurospheres. (**B**) BF image of the iMuSC-formed neurospheres in suspension. (**C–F**) Immunofluorescence images of cryosectioned neurospheres after 7 days of culture. They stained positive for CNPase (red, **C**), Nestin (green, **D**), Nefm (red, **E**), and Gfap (green, **F**). Nuclei were stained with DAPI (blue). Scale bar = 100 µm. (**F)** Whisker plots summarize the gene expression profiles of five biological replicates of iMuSC-formed neurospheres after 7 days of culture compared to undifferentiated iMuSCs by qPCR.

For neuronal differentiation, undifferentiated iMuSCs were transferred as single cells into NSC medium and cultured for 1 week. Within this time, iMuSCs formed neurosphere-like structures floating in suspension (Fig. 2B), while the control MuSCs showed no sign of forming these structures (Fig. 2A). These spheres stained positive for the early neuronal progenitor/stem cell marker Nestin (Fig. 2D), and for some further differentiated neural phenotypes such as oligodendrocytes by (i.e., 2′,3′-cyclic-nucleotide 3′-phosphodiesterase [CNPase] (Fig. 2C), neurons for by the neuronal cytoskeleton marker Neurofilament-M (Nefm) (Fig. 2E), and astrocytes by glial fibrillary acidic protein (Gfap) (Fig. 2F). Gene expression profiles of the iMuSCs-induced neurospheres were also analyzed by qPCR. As confirmation of the immunocytochemistry results, neuronal and neuronal progenitor/stem cell markers, such as Nestin, Nefm and β-Tubulin III, and other lineage markers (i.e., CNPase, Gfap, and Orthodenticle homeobox 2 [Otx2]) were highly overexpressed in the iMuSC-formed neurospheres compared to the undifferentiated iMuSCs (Fig. 2G).

**Figure 3:**
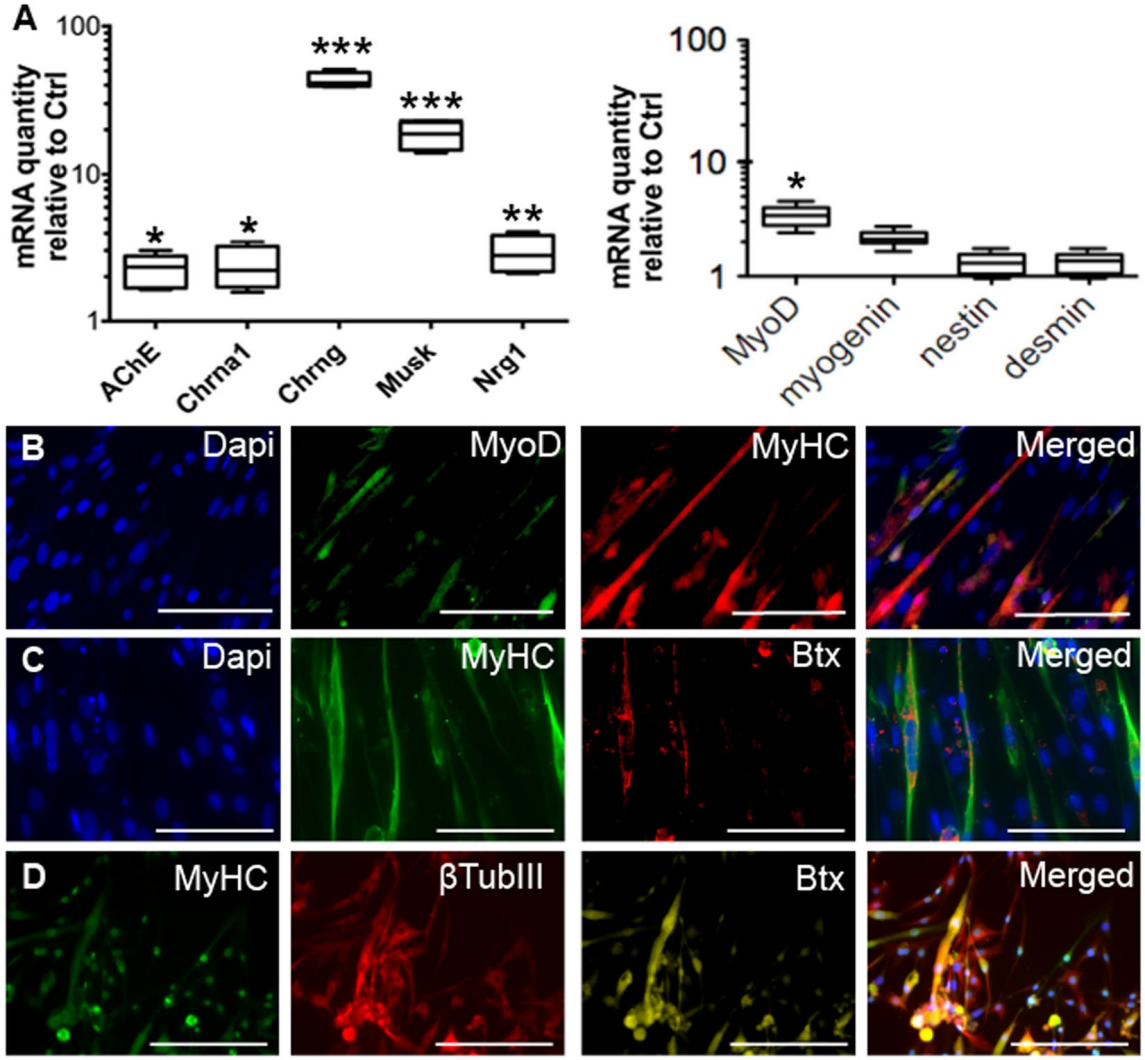
Myogenic differentiation of iMuSCs and NMJ formation. (**A**) Whisker plots summarize the expression of NMJ-related genes as well as the myogenic genes within the neurosepheres in culture, analyzed by qPCR. (**B**) After the neurospheres were replaced into monolayer culture in MD Medium, their recovered their myogenic ability, formed myotube-like structures, and begun expression of myogenic protein MyoD (green, **B**), MyHC (Red, B; green, **C**,**D**). The attached neurospheres also formed neuron-like structures expressing β-Tubulin III (red, **D**). The presence of NMJs was shown by Btx staining (red, **C**; yellow, **D**). Nuclei were stained with Dapi. Scale bar = 100 µm.

The iMuSC-formed neurospheres were harvested and plated in collagen type IV/poly-L-ornithine/laminin coated 24-well tissue culture plates (Corning, 2–3 neurospheres/well) in MD medium in 5% CO_2_ at 37 °C for 14 days. In these conditions, iMuSC-formed neurospheres proliferated to near-confluence and aligned in preparation for fusion, and extensive myotube formation was observed with MyoD and Myosin heavy chain (MyHC) expression (Fig. 3B–D, and Suppl. Movie). Interestingly, the attached neurospheres also formed neuron-like structures, expressing β-Tubulin III (Fig. 3D). The presence of formed NMJs was demonstrated by α Bungarotoxin (Btx) staining (Fig. 3C,D). In addition, several NMJ-related genes were overexpressed, but myogenic genes were expressed in a low level in the culture as demonstrated by qPCR analysis in these neurospheres, including genes with NMJs: acetylcholinesterase (AChE), cholinergic receptors (nicotinic, alpha 1 [Chrna1], and gamma [Chrng], muscle-specific kinase (MuSK), neuregulin 1 (Nrg1), and myogenic genes: MyoD, and Myogenin (Fig. 3A).

### Monolayer differentiation of iMuSCs along the neuronal pathway

We tested several culture conditions to induce neuronal differentiation of iMuSCs. In our laboratory, the attachment of the neurospheres and the appearance of cells with neuronal morphology were limited and the plated cells did not survive more than 1 week using previously published protocols. In order to improve the attachment and neuronal differentiation of iMuSCs, we tested 2 additional media (Suppl. Table 3). The percentages of attached iMuSC-induced neurospheres on day 7 after plating were 17% in ND1 media, 75% in ND2 media, and 92% in ND3 media. For further experiments, we used the ND3 medium in which the attached neurospheres could mature and form neuron-like structures on collagen type IV/polyornithine/laminin coated plates.

**Figure 4:**
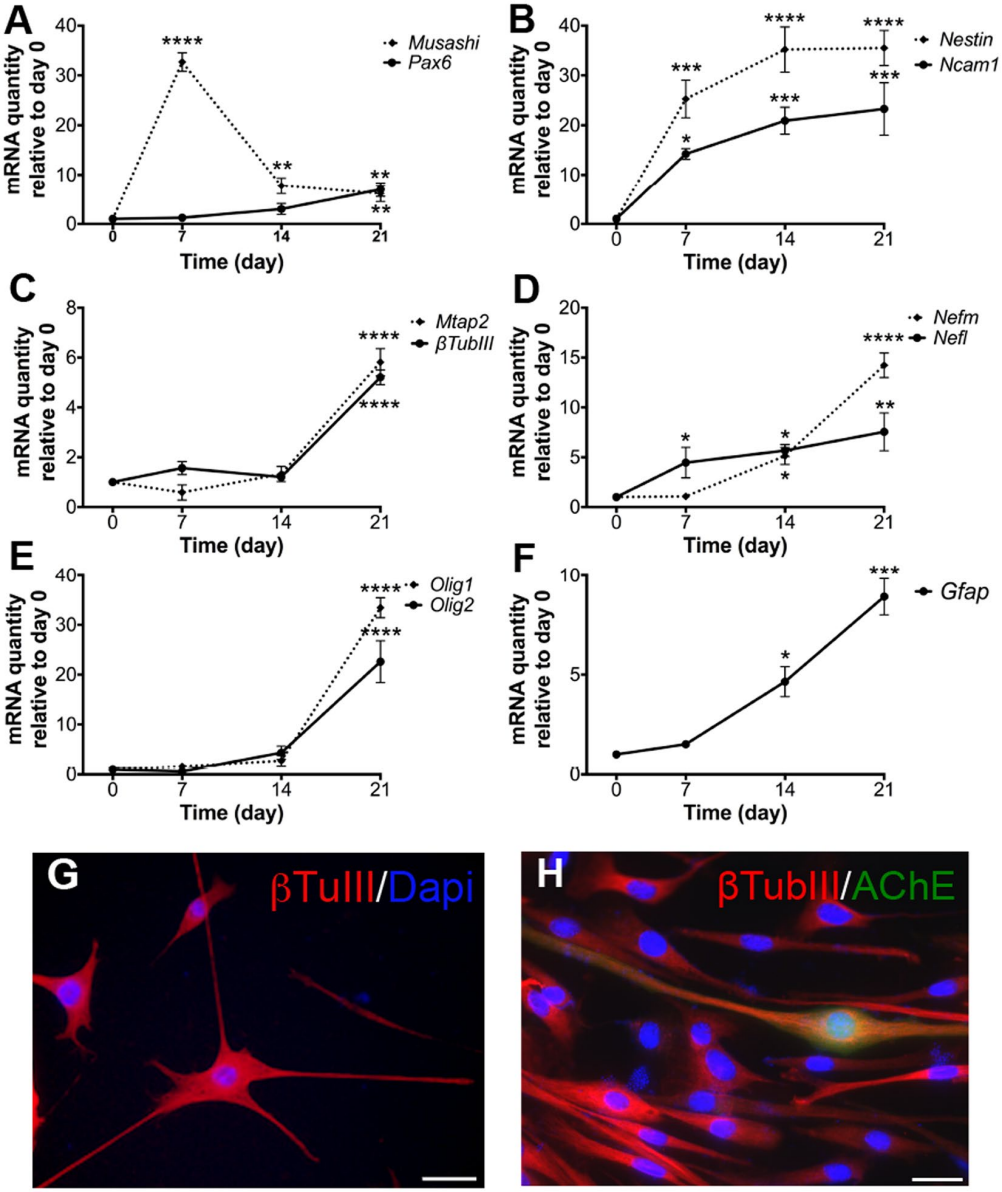
Induced neuronal differentiation of iMuSCs. (**A–F**) Gene expression kinetics were analyzed by qPCR during the 21-day neuronal differentiation period of the plated iMuSC-formed neurospheres. Data are presented as ± SEM of five biological replicates. *P < 0.05, **P < 0.01, ***P < 0.001, ****P < 0.0001. (**G**,**H**) Immunocytochemical staining of neurons after 21-day differentiation from iMuSC-formed neurospheres. β-Tubulin III (red, **G**,**H**) and AChE (green, **H**). Nuclei were stained with DAPI. Scale bar = 100 µm (**G**) and 10 µm (**H**).

Real-time PCR experiments confirmed the appearance of cells expressing neuronal marker genes and revealed gene expression kinetics during neuronal precursor differentiation of iMuSCs (Fig. 4). The expression of each gene was analyzed in samples from undifferentiated iMuSCs (day 0) as well as from days 7, 14, and 21. There were no statistically significant differences in the expression of the housekeeping gene Gapdh between the different time points of sampling (P > 0.05).

The Musashi RNA-binding protein 1, the adhesion molecule Ncam1, the intermediate filament Nestin, and the transcription factor Pax6 are expressed in neuronal precursors in neuroepithelium *in vivo* ^25–27^. Musashi1 (Fig. 4A) was strongly upregulated (32.7 ± 3 fold) on day 7 of differentiation. During the neuronal maturation process, Musashi expression was downregulated (6.1 ± 2 fold) on day 21. A significant (8.4 ± 1 fold) upregulation of Pax6 (Fig. 4A) mRNA expression was demonstrated on day 21. This upregulation was initiated already on day 7 (1.2 ± 0.5 fold), although it was not statistically significant at this time point (P > 0.05). Nestin (Fig. 4B) mRNA expression was increased already from day 7 (25.3 ± 3 fold) and stayed at a plateau (35.2 ± 2 fold) for the later time points investigated. Ncam1 (Fig. 4B) mRNA expression continuously increased during the 21 days of differentiation and was significantly different to control levels at day 7 (14.2 ± 2 fold), 14 (20.9 ± 3 fold) and 21 (23.3 ± 4 fold). Microtubule-associated protein Mtap2, β-Tubulin III, Nefm and Nefl are neuronal cytoskeletal proteins expressed in mature neurons^28–30^. Expression of mRNA for Mtap2 and β-Tubulin III (Fig. 4C) was upregulated on day 21 of differentiation, showing 5.8 ± 1 and 5.2 ± 0.5 fold increase, respectively. Neither Mtap2 nor β-Tubulin III mRNA expression was significantly changed (*P > *0.05) on days 7 and 14 as compared to undifferentiated iMuSCs (day 0). Expression of mRNA for Nefm and Nefl (Fig. 4D) was showing a continuous upregulation from day 7 on, with 2.1 ± 0.3 and 4.9 ± 0.5 fold, reaching a 7.2 ± 06 and 14.5 ± 1 fold increase at day 21. The basic helix-loop-helix transcription factors, oligodendrocyte transcription factor 1 (Olig1) and Olig 2 are early oligodendrocyte progenitors that are coordinately expressed in the developing central nervous system and postnatal brain determining oligodendrocyte differentiation *in vivo* ^31^. A strong upregulation of Olig1 (33.4 ± 2 fold) and Olig2 (22.6 ± 3 fold) mRNA expression level was shown on day 21 of differentiation (Fig. 4E). The upregulation seemed initiated on day 14 (2.7 ± 1 fold), although it was not statistically significant. Gfap is an intermediate filament protein that is expressed mainly by astrocytes in the central nervous system^32^. Expression of mRNA for Gfap was upregulated at days 14 and 21 of differentiation, showing 6.6 ± 1 and 8.9 ± 1 fold increase compared to undifferentiated iMuSCs (Fig. 4F).

Immunocytochemical analysis supported the qPCR data on neuronal marker expression. Neuronal precursor differentiation resulted in appearance of mature, elongated cells on day 21 which expressed antigens for antibodies against the neuronal cytoskeletal proteins β-Tubulin III (Fig. 4G,H) and AChE (Fig. 4H).

### Mature and functional neuronal development of iMuSCs *in vitro*

**Figure 5:**
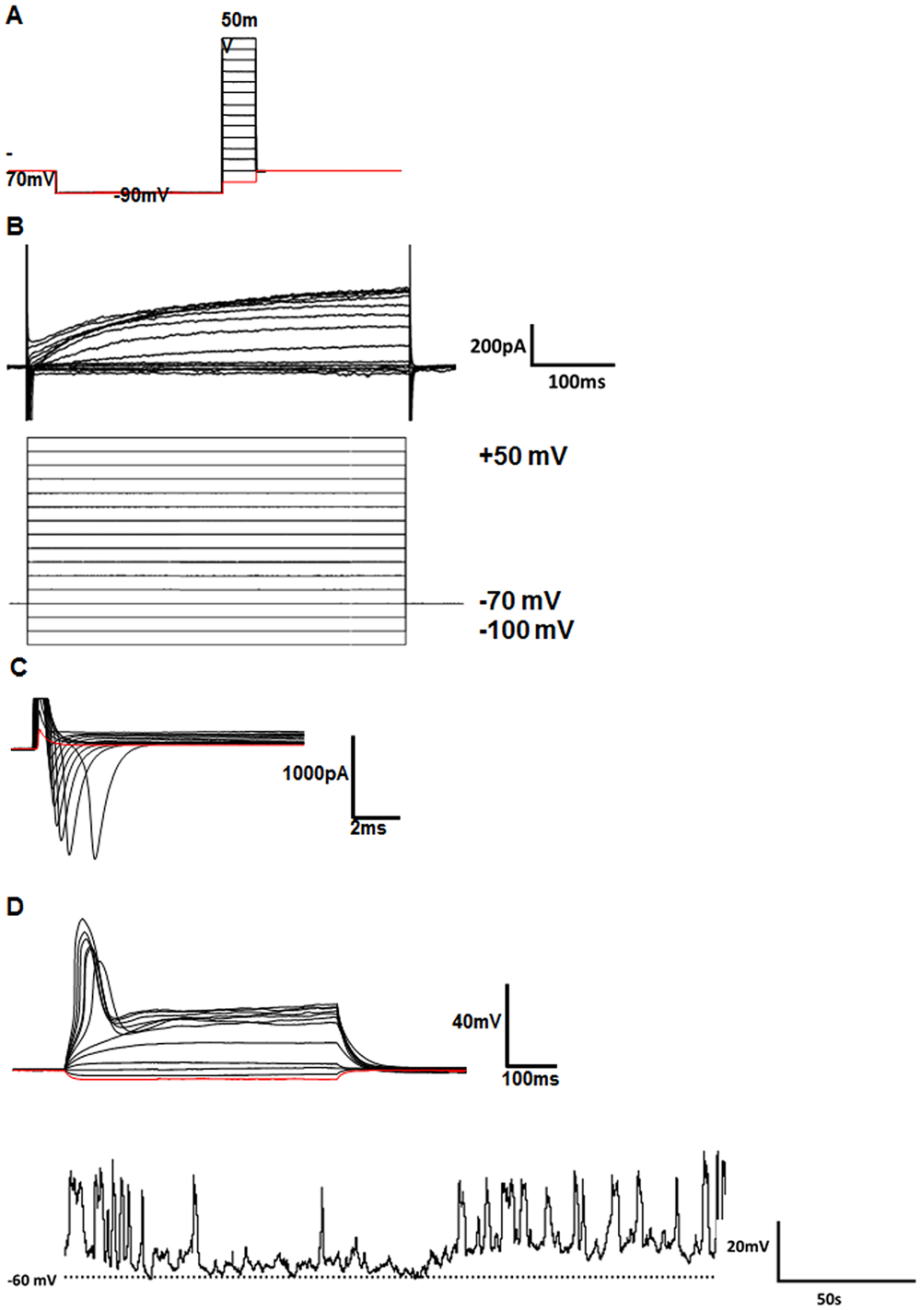
Electrophysiological evidence for successful neuronal development. (**A**) Whole-cell voltage clamp recording protocol used for patch-clamp analysis. (**B**) Representative example for K^+^ currents observed during 500 ms voltage steps of the whole-cell voltage clamp recording protocol displayed in (**A**). (**C**) Representative example of Na^+^ currents observed during 20-ms voltage steps of the whole-cell voltage clamp recording protocol displayed in (**A**). Note that Na^+^ currents (downward deflections) were observed at voltages ≤−30 mV. (**D**) Individual traces of action potential-like events generated by an intracellular current injection protocol. Spontaneous action potential-like events were recorded in current-clamp mode (0 pA). The dashed line indicates the −60 mV resting membrane potential.

The electrophysiological properties of neurons from at least three independent differentiation passes were evaluated using voltage- and current-clamp recordings. iMuSC-induced neurospheres were differentiated on Poly-L-ornithine/Laminin coated glass coverslips towards the neuronal lineage for 21 days and then placed into a recording chamber for whole-cell patch-clamp studies. Differentiation into mature, electrophysiologically active neurons was shown by the presence of voltage-dependent Na^+^ and K^+^ currents and spontaneous action potentials in individual patch-clamped neurons (Fig. 5). Thus, our differentiation protocol yielded *bona fide* neurons from iMuSCs.

### Improved NMJ morphology by transplanted iMuSCs or iMuSC extracts in mdx mice

**Figure 6:**
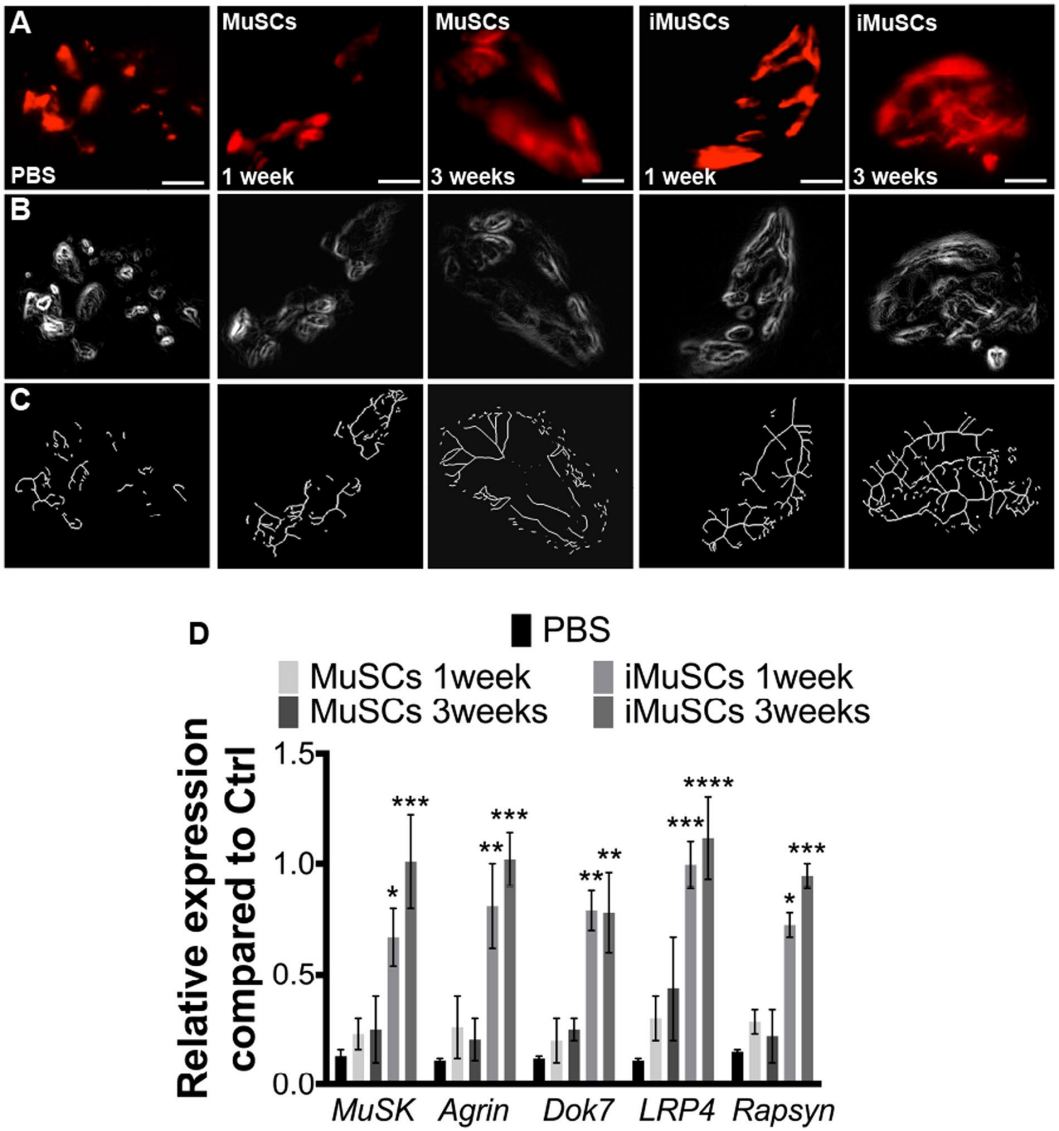
NMJ recovery after transplantion of iMuSCs into *mdx* mice. (**A**) Representative IF images of NMJs after treatment of GMs with no cells, MuSCs, and iMuSCs in *mdx* mice (red, **A**). (**B**) Processed binary images and (**C**) skeletonized images show discontinuity and branching patters. (**D**) Expression of MuSK complex constituents. Data are presented as mean ± SEM of five biological replicates. *P < 0.05, **P < 0.01, ***P < 0.001, ****P < 0.0001.

**Figure 7:**
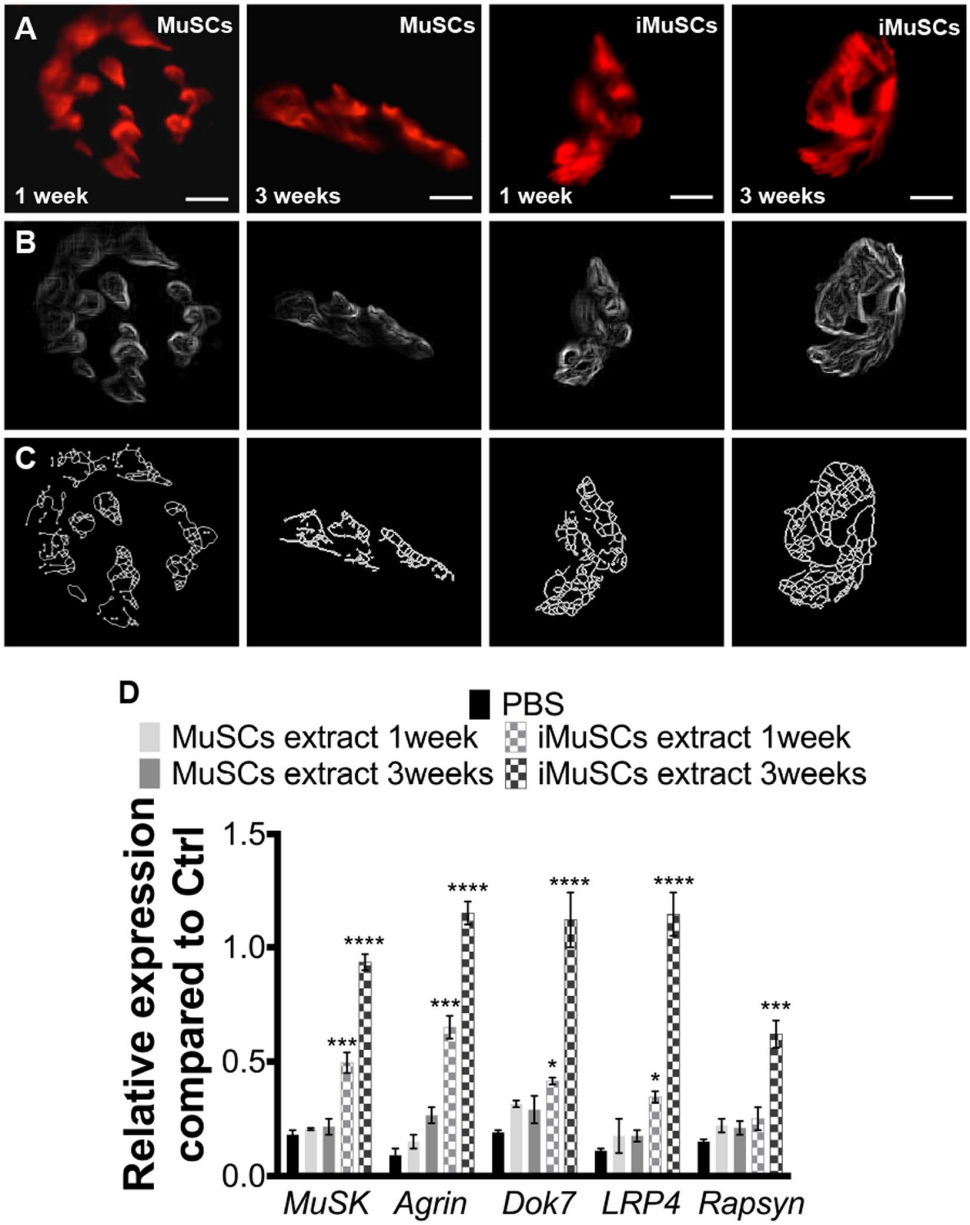
NMJ recovery after injection of iMuSC extracts into *mdx* mice. (**A**) Representative IF images of NMJs after injection with MuSC and iMuSC extracts into the GMs of *mdx* mice (red, **A**). (**B**) Processed binary images and (**C**) skeletonized images show discontinuity and branching patters. (**D**) Expression of MuSK complex constituents. Data are presented as mean ± SEM of five biological replicates. *P < 0.05, **P < 0.01, ***P < 0.001, ****P < 0.0001.

Since the iMuSC-formed neurospheres displayed heterogeneous phenotypes, they were not suitable for cell therapy applications. Therefore, undifferentiated iMuSCs and control MuSCs were separately injected into the left and right GM muscles of *mdx* mice. *Mdx* muscle treated with control PBS and MuSCs showed a non-continuous, punctate pattern of NMJ morphology; however, the NMJ morphology was markedly improved in the iMuSC-treated muscles (Fig. 6A–C), and also in the iMuSC extract-treated muscles (Fig. 7A–C). These morphological changes were quantified by the number of segments and the means of terminal and branching nodes (Suppl. Table 4). Based on these data, the acetylcholine receptors (AChRs) in *mdx* mice were discontinuous and punctate, consistent with previous findings in recent literature^33, 34^. However, after iMuSC transplantation, AChRs in *mdx* mice showed a significantly improved morphology as evidenced by more dense aggregates, increased continuity and greater number of segments, and the presence of terminal nodes and branching nodes. These differences indicate a transient improvement in AChRs density due to the transplantation of iMuSCs and iMuSC -extracts into the muscle tissues of *mdx* mice.

Muscle-specific kinase (MuSK) is a transmembrane tyrosine kinase crucial for forming and maintaining the NMJs; activation of the MuSK complex drives AChR clustering^35, 36^. It has been previously shown that expression of the MuSK complex is altered in *mdx* mice^37^. Transcripts for the multiprotein MuSK signaling complex (Agrin, LRP4, Dok7, MuSK and Rapsyin) were obtained from five 6–8 week-old male wild-type *C57BL/6J* mice and five 6–8-week-old male control, and MuSCs and iMuSCs (along with their respective extracts) -injected *mdx* mice were analyzed by qPCR (Figs 6D and 7D). Gene expression levels were compared to the control wild-type *C57BL/6J* mice. Interestingly, in contrast to previously-published results^37^, we found that the levels of mRNA expression for each constituent of the MuSK complex was significantly decreased in the *mdx* mice and their expression was markedly improved 3 weeks after the injection of iMuSCs and iMuSC extract.

## Discussion

Complex biological processes occur following skeletal muscle injury and repair. Several time-dependent phases are interrelated with healing, including muscle degeneration/necrosis, inflammation/immune responses, stem cell activation, tissue regeneration/remodeling, and functional repair with the deposition of fibrous scar tissue^38, 39^. Functional repair includes the complete reinnervation and revascularization in the regenerated muscle fibers. Although the process of identifying innervation is challenging, due to difficulties in tracking the appropriate biomarkers, newly formed NMJs between the surviving axons and the regenerated muscle fibers have been identified 2 weeks after skeletal muscle injury^40^. Previous reports have indicated that MuSCs could also give rise to neuronal-like cells, based on the expression of the neuron-associated genes or proteins^15, 41^, suggesting that muscle stem cells could potentially differentiate into the lineage of neighboring cells. These studies discuss the multipotency or transdifferentiation potential of mesenchymal stem cells (MSCs); however, this turned out to be due mostly to *in vitro* assays reflecting the potential of the culture medium rather than the potential of the cells^42–44^. Thus, *in vivo* cell implantation studies are extremely important to prove the concept of the multipotent differentiation ability of iMuSCs.

We are not aware of any other published reports that demonstrate the existence of a side population of stem cells within injured skeletal muscle tissue that can differentiate into functional neurons *in vitro* and improve NMJs formation *in vivo*. Our qPCR results following iMuSC neuronal differentiation provide detailed information on gene expression kinetics for this unique stem cell population. Expression of mRNA markers for neuronal precursors (Musashi, Ncam1, Nestin, and Pax6) and for mature neurons (Mtap2, β-Tubulin III, Nefm, and Nefl) were increased throughout the process of neuronal differentiation; several of these markers were significantly upregulated (e.g. Pax6, Ncam1, Nefm, and Mtap2). The sequence of neuronal differentiation events (such as the formation of neuronal precursors and their further maturation,) was reflected in their mRNA expression profiles and followed similar patterns and temporal windows as those that took place during neurogenesis. Additionally, our differentiation protocol gave rise to a small population of oligodendrocytes and astrocytes as evidenced by the upregulated mRNA expression of Olig1, Olig2, and Gfap. Our immunocytochemical analyses supported this qPCR data by demonstrating the presence of β-Tubulin III, Nefm, and AChE. To confirm the successful neuronal differentiation of iMuSCs, whole-cell patch-clamp studies demonstrated the presence of voltage-dependent Na^+^ and K^+^ currents and spontaneous action potentials within individual patch-clamped neurons. Although these electrophysiological findings may not have represented the entire culture, our data from iMuSC-differentiated neurons were qualitatively similar to previously published functional data obtained from neurons generated from mouse embryonic stem cells^45, 46^. Altogether, these data suggest the appearance of mature and functional neuronal-like cells derived from the differentiation of iMuSCs.

We selected a DMD mouse model to investigate the processes of NMJ formation and function. DMD is a relatively common X-linked neurodegenerative disorder in which progressive muscle degeneration, weakness, increased susceptibility to muscle injury, and inadequate repair underlie its pathologic features; the disorder affects approximately 1 in 3,200 male infants worldwide^47, 48^. In normal mammalian muscle, AChRs densely cluster in winding, band-like arrays on post-synaptic membranes^49^. These bands most often form a continuous structure commonly referred to as having a “pretzel-shape”. To achieve maximally efficient neurotransmission, pre- and post-synaptic membranes exhibit the same band pattern. In patients with DMD, however, this “pretzel-shape” is fragmented into many individual gutters^49–51^. The causes and molecular events resulting in fragmentation of the NMJs in DMD patients is still unclear. Although current published studies agree that the primary defect of DMD is confined to the stability of the muscle cell membrane^52^, a detailed understanding the biomolecular sequences leading to NMJ dysfunction and its contribution to the functional deficits observed in DMD could revolutionize the treatment of many types of musculoskeletal conditions.

Multifunctional signaling complexes are involved in the organization of NMJs^35^. MuSK is a transmembrane tyrosine kinase crucial for forming and maintaining the NMJs^36^. Activation of the MuSK complex drives AChR clustering. Based on the fact that mutations of MuSK complex are responsible for diseases involving the NMJ, it would be interesting to determine whether the upregulation of MuSK in dystrophic muscle would rescue the deterioration of its NMJs. Our results confirmed that NMJ morphology was transformed in the muscles of *mdx* mice and that the levels of MuSK-related gene expression were also altered. These results contrast those of a previous study in which decreased MuSK-related mRNA levels represented the only significant differences between wild-type and *mdx* muscles; the levels of mRNA expression of Agrin, LRP4, Dok7, and Rapsyn were not changed^37^. Furthermore, in order to verify the neurogenic potential of iMuSCs, we transplanted undifferentiated iMuSCs or iMuSCs extracts into the GM muscles of *mdx* mice. Three weeks after the transplantation, we observed significant improvements in NMJs morphology (i.e., more dense aggregates, increased continuity, greater numbers of segments, and the presence of terminal and branching nodes), which corroborated with the increased mRNA expression level of the MuSK complex when compared to the control MuSCs, indicating the essential role of iMuSCs in improving NMJs in dystrophic muscle.

In summary, iMuSCs isolated from the injured murine skeletal muscles has a remarkable efficiency to form mature and functional neurons and has significant benefits to the restoration of NMJ function within those injured or diseased skeletal muscles *in vivo*. According to our results, we hypothesize that the observed beneficial effects of intramuscular iMuSC transplantation are due in large part to the variety of growth factors, chemokines, cytokines, immunosuppressive molecules, among many other factors secreted in physiologic ratios by iMuSCs at the site of injury. In future studies, elucidating the roles iMuSCs-derived biomolecules for tissue regeneration may provide insight into the development of alternative therapies that can overcome current cell sourcing issues, especially in the treatment of neuromuscular conditions.

## Electronic supplementary material

Supplementary Information

Contraction of myotubes

Supplementary legend of Video
