# Supplementary material for: Functional Neuronal Differentiation of Injury-Induced Muscle-Derived Stem Cell-Like Cells with Therapeutic Implications
Source: Sci Rep. 2017 Apr 26;7:1177. doi: 10.1038/s41598-017-01311-4 (PMC5430871; doi:10.1038/s41598-017-01311-4)
Supplement: Supplementary file 1 — Supplementary Information [file 41598_2017_1311_MOESM1_ESM.pdf]

# Functional Neuronal Differentiation of Injury-Induced Muscle-Derived Stem Cell-Like Cells with Therapeutic Implications*

**Authors:** Kinga Vojnits[1,2], Haiying Pan[1,3], Xiaojing Dai [1,2], Hao Sun[4], Qingchun Tong[4], Radbod Darabi[1], Johnny Huard[3,5,6], Yong Li[1,2,6]

***Affiliations:*** *1. Center for Stem Cell and Regenerative Medicine, The Brown Foundation Institute of Molecular Medicine for the Prevention of Human Diseases (IMM) at the University of Texas Health Science Center at Houston, TX, USA; 2. Department of Pediatric Surgery, University of Texas McGovern Medical School at Houston, USA; 3. Department of Orthopeadic, University of Texas McGovern Medical School at Houston, USA; 4. Center for Metabolic and Degenerative Disease, The IMM at the University of Texas Health Science Center at Houston, TX, USA; 5. Center for Sports Regenerative Medicine, Steadman Philippon Research Institute, Vail, CO, USA; 6. Center for Tissue Engineering and Aging Research, The IMM at the University of Texas Health Science Center at Houston, TX, USA.*

**Travel award for K.V., The American Society for Neural Therapy and Repair (ASNTR), April 24-26, 2014. Tampa, Florida, USA.*

**Correspondence information for corresponding author:**

Yong Li, M.D. Ph.D.

1825 Pressler Street, Houston, TX 77030, USA

+1 (713) 500-2438

Yong.Li.1@uth.tmc.edu

**Key words:** *Skeletal muscle injury, stem cells, neuronal differentiation, mature functional neurons, neuromuscular junctions (NMJs).*

**Supplementary Table 1: Primary and secondary antibodies.**

| Primary antibodies | Host | Dilution | Manufacturer | Secondary antibodies |
|---|---|---|---|---|
| AChE | Mouse monoclonal IgG2b | 1:200 | Abcam | Goat anti-mouse AlexaFluor 488 |
| CNPase | Mouse monoclonal IgG1 | 1:200 | Millipore | Goat anti-mouse AlexaFluor 594 |
| Gfap | Rabbit polyclonal IgG | 1:150 | Santa Cruz | Goat anti-rabbit AlexaFluor 488 |
| Nefm | Mouse monoclonal IgG2a | 1:200 | Abcam | Goat anti-mouse AlexaFluor 594 |
| Nestin | Goat polyclonal IgG | 1:150 | Santa Cruz | Donkey anti-goat AlexaFluor 488 |
| Myosin heavy chain | Mouse monoclonal IgG1 | 1:200 | Sigma | Goat anti-mouse AlexaFluor 488 |
| β-Tubulin III | Rabbit plyclonal IgG | 1:500 | Abcam | Goat anti-rabbit AlexaFluor 594 |

**Supplementary Table 2: PCR primers.**

| Gene | 5'Primer | 3'Primer |
|---|---|---|
| **AChE** | AAGGGCTGGGATATAATACGAC | CTTAGCCCAAGACATGCAGA |
| **Agrin** | CCTCAACTTGGACACGAAGCT | AGGCCGATGCCCACAGA |
| **β-Actin** | GGCTGTATTCCCCTCCATCG | CCAGTTGGTAACAATGCCATGT |
| **Chrna1** | GAATCCAGATGACTATGGAG | GACAATGATCTCACAGTAGC |
| **Chrng** | ATCCGGCACCGACCGGCTAA | CATTTCTGCCCGCCCGCCTT |
| **Dok7** | TCTCCCAGACCCGAGTTCTG | TCTAGCTGCAGGGCTTCCA |
| **Gapdh** | AACTTTGGCATTGTGGAAGG | GGATGCAGGGATGATGTTCT |
| **Gfap** | GCCCGGCTCGAGGTCGAG | GTCTATACGCAGCCAGGTTGTTCT CT |
| **Lrp4** | GGACTGCACGTCAGCTATGC | CGCGATCACCAACAAAATCA |
| **Musashi** | GTTCATCGGAGGACTCAG | GCTCTCAAACGTGACAAA |
| **Musk** | TGAGAACTGCCCCTTGGAACT | GGGTCTATCAGCAGGCAGCTT |
| **Mtap2** | ACACCCCGAACCAGGAGGA | GCGTTGGACGTGCCCTTCT |
| **Ncam1** | ATGGAAACTCTATTAAAGTGAAC CTGA | TAGACCTCATACTCAGCATTCCAG T |
| **Nefm** | AGTGGTTCAAATGCCGCTAC | TTTTCCAACTGCTGGATGGT |
| **Nefl** | CCATGCAGGACACAATCAAC | CGCCTTCCAAGAGTTTTCTG |
| **Nestin** | CTGGAAGGTGGGCAGCAACT | ATTAGGCAAGGGGGAAGAGAAGG ATG |

| | | |
|---|---|---|
| **Nrg1** | CCTGGGAGGCCCTCGCGAAT | CCGTCATGCTGGACACGGGT |
| **Olig1** | ACGTCGTAGCGCAGGCTTAT | CGCCCAACTCCGCTTACTT |
| **Olig2** | GGGAGGCGCCATTGTACA | GTGCAGGCAGGAAGTTCCA |
| **Otx2** | CTGTTACCAGCCATCTCAATC | ATAGCTTCTACAGGTCTTCAC |
| **Pax6** | AGGGGGAGAGAACACCACT | CATTTGGCCCTTCGATTAGA |
| **Rapsyn** | ACGAGTGCGTGGAGGAGACT | TGTTCCTCTCCCCGATGGA |
| **β-TubulinIII** | AGACAACTTCGTTTTCGGTCAGT | CCTTTAGCCCAGTTGTTGCCT |

**Supplementary Table 3: Tested medium for neuronal differentiation of iMuSCs.**

| | |
|---|---|
| **ND1 medium** | DMEM/F12, 1% Glutamax, 1% non-essential amino acids, 0.1mM β-mercaptoethanol, 1% ITS (5 μg/ml insulin, 5 μg/ml transferring, 29nM selenium), 20nM progesterone, 100 μM putrescine dihydrochloride, 1 μg/ml laminin, 5 ng/ml human recombinant basic fibroblast growth factor, 0.5% antibiotics |
| **ND2 medium** | Neurobasal Medium, 1% Glutamax, 2% B-27, 0.5% antibiotics |
| **ND3 medium** | Neurobasal Medium, 1% Glutamax, 1% B-27, 20ng/ml brain-derived neutrophic factor, 0.5% antibiotics |

**Supplementary Table 4: Morphological characteristics of the neuromuscular junctions.**

| | Terminal Nodes | Branching Nodes | Nb of Segments | Total Length |
|---|---|---|---|---|
| **PBS** | 79±7 | 13±3 | 59±6 | 1135.5±87 |
| **MuSCs 1week** | 83±6 | 21±2 | 49±3 | 1205.3±110 |
| **MuSCs 3weeks** | 85±8 | 28±3 | 63±5 | 1167.3±78 |
| **iMuSCs 1week** | 80±8 | 31±5 | 95±9* | 1879.4±98* |
| **iMuSCs 3weeks** | 144±11* | 58±4* | 163±1* | 3099.7±112* |
| **MuSCs extract 1week** | 86±8 | 10±2 | 45±2 | 995.34±98 |
| **MuSCs extract 3weeks** | 89±5 | 18±6 | 53±8 | 1058.4±82 |
| **iMuSCs extract 1weeks** | 89±8 | 45±9* | 104±97* | 2920.3±101* |
| **iMuSCs extract 3weeks** | 135±13* | 63±7* | 142±12* | 3233.5±125* |

All data are presented as mean ±SEM, $p < 0.05$. ***,** indicates statistical significance from control PBS injected *mdx* mice
