# Supplementary material for: Functional Neuronal Differentiation of Injury-Induced Muscle-Derived Stem Cell-Like Cells with Therapeutic Implications
Source: Sci Rep. 2017 Apr 26;7:1177. doi: 10.1038/s41598-017-01311-4 (PMC5430871; doi:10.1038/s41598-017-01311-4)
Supplement: Supplementary file 3 — Supplementary legend of Video [file 41598_2017_1311_MOESM3_ESM.pdf]

Functional Neuronal Differentiation of Injury-Induced Muscle-Derived Stem Cell-Like Cells with Therapeutic Implications*

Authors: Kinga Vojnits[1,2], Haiying Pan[1,3], Xiaojing Dai [1,2], Hao Sun[4], Qingchun Tong[4], Radbod Darabi[1], Johnny Huard[3,5,6], Yong Li[1,2,6]

Affiliations: *1. Center for Stem Cell and Regenerative Medicine, The Brown Foundation Institute of Molecular Medicine for the Prevention of Human Diseases (IMM) at the University of Texas Health Science Center at Houston, TX, USA; 2. Department of Pediatric Surgery, University of Texas McGovern Medical School at Houston, USA; 3. Department of Orthopeadic, University of Texas McGovern Medical School at Houston, USA; 4. Center for Metabolic and Degenerative Disease, The IMM at the University of Texas Health Science Center at Houston, TX, USA; 5. Center for Sports Regenerative Medicine, Steadman Philippon Research Institute, Vail, CO, USA; 6. Center for Tissue Engineering and Aging Research, The IMM at the University of Texas Health Science Center at Houston, TX, USA.*

**Travel award for K.V., The American Society for Neural Therapy and Repair (ASNTR), April 24-26, 2014. Tampa, Florida, USA.*

Correspondence information for corresponding author:

Yong Li, M.D. Ph.D.

1825 Pressler Street, Houston, TX 77030, USA

+1 (713) 500-2438

Yong.Li.1@uth.tmc.edu

Key words: *Skeletal muscle injury, stem cells, neuronal differentiation, mature functional neurons, neuromuscular junctions (NMJs).*

Spontaneous contraction of iMuSCs-formed myotubes in vitro

The iMuSC-formed neurospheres were harvested and plated in collagen type IV/poly-L-ornithine/laminin coated 24-well tissue culture plates in muscle differentiation medium (DMEM supplemented with 2% horse serum and 1% Penicillin/Streptomycin antibiotics) in 5% CO_2 at 37°C for 14 days. In these conditions, iMuSC-formed neurospheres proliferated to near-confluence and aligned in preparation for fusion, and extensive myotube formation was observed. The spontaneous contractions of myotubes were recorded with a time-lapse microscope. Approximately 70% of the myotubes were observed to contract spontaneously, and the contractions lasted for at least one hour without any further stimulation.
